# Advancing aged care: a systematic review of economic evaluations of workforce structures and care processes in a residential care setting

**DOI:** 10.1186/s12962-016-0061-4

**Published:** 2016-12-12

**Authors:** Tiffany Easton, Rachel Milte, Maria Crotty, Julie Ratcliffe

**Affiliations:** 1 Flinders Health Economics Group, Flinders University, Adelaide, Australia; 2 NHMRC Partnership Centre on Dealing with Cognitive and Related Functional Decline in Older People, University of Sydney, Sydney, Australia; 3 Rehabilitation, Aged and Extended Care, Flinders University, Adelaide, Australia

**Keywords:** Systematic review, Long-term care, Economic evaluation

## Abstract

Long-term care for older people is provided in both residential and non-residential settings, with residential settings tending to cater for individuals with higher care needs. Evidence relating to the costs and effectiveness of different workforce structures and care processes is important to facilitate the future planning of residential aged care services to promote high quality care and to enhance the quality of life of individuals living in residential care. A systematic review conducted up to December 2015 identified 19 studies containing an economic component; seven included a complete economic evaluation and 12 contained a cost analysis only. Key findings include the potential to create cost savings from a societal perspective through enhanced staffing levels and quality improvement interventions within residential aged care facilities, while integrated care models, including the integration of health disciplines and the integration between residents and care staff, were shown to have limited cost-saving potential. Six of the 19 identified studies examined dementia-specific structures and processes, in which person-centred interventions demonstrated the potential to reduce agitation and improve residents’ quality of life. Importantly, this review highlights methodological limitations in the existing evidence and an urgent need for future research to identify appropriate and meaningful outcome measures that can be used at a service planning level.

## Background

The United Nations has reported population ageing in nearly every country in the world and projections suggest that the number of people aged 60 and over will more than double the 2013 level by 2050 [1]. Even greater will be the expected growth in the so-called ‘oldest old’ or those aged 80 years and older, with the population in this age group expected to rise from 4 to 10% of the population [2]. Two likely consequences of the ageing population will be an increase in the prevalence of dementia and a growing demand for residential aged care. Dementia prevalence increases dramatically with age from roughly 3% in those aged 70–74 to over 20% for those aged 85 and over [3]. Expert consensus estimates the number of people living with dementia will almost double every 20 years, reaching over 81 million people worldwide by 2040 [3].

Aged care is a significant responsibility for governments. In most OECD countries, aged care accounts for roughly 1–1.5% of GDP in terms of government funding [4], and on average roughly two-thirds of this funding is allocated to residential care [5]. The proportion of the population receiving long-term care has also grown, rising to 2.3% of the population in OECD countries in 2013 [2]. Given the high prevalence of use of these services among older people, especially the rapidly growing ‘oldest old’, the need for these services is expected to continue to grow, although to what extent is likely to depend upon the health status of individuals as they age, the presence of dementia, as well as other social trends, such as the ability of family members to provide informal care. It is estimated that over 50% of residents residing in residential aged care facilities have a recorded diagnosis of dementia [6–9], and thus it is imperative for people with dementia to be included in research studies conducted in this setting. Several recent studies have indicated that for people with dementia with high levels of physical dependence, residential care can be less costly to provide than home-based care [10–12].

Residential care is in the midst of a ‘culture change’ movement, involving organisational change and a move toward providing more person-centred, individualised care [13]. Person-centred care is also increasingly being recognised as an important focus for the care of individuals living with dementia. A social-psychological theory of dementia care, developed by Kitwood and Bredin [14], links agitation to negative contextual stimuli that neglect personhood. According to the theory, warm and compassionate care interactions should increase well-being, while disrespectful and disengaged care interactions are thought to lead to decreased well-being and increased agitation. Questions remain, however, as to the optimal implementation approaches and staffing configurations to achieve a high quality residential care experience for residents.

The framework of economic evaluation is increasingly being applied in health and aged care services in an effort to promote efficiency in the design and delivery of services. Knowledge of the incremental costs and effectiveness of differing program design features is essential for well-informed resource allocation decisions in residential care. Program design features can be broken down into subcategories to assist in the assessment of quality (see Donabedian [15]). This review focuses on the economic evidence of program features which directly relate to how care is provided in terms of the workforce and its operations (structures of care) and the services provided (processes of care).

To this end, the main objectives of this review were to answer the following questions:

1. Which structures and processes in residential aged care settings have been demonstrated to be cost effective?
2. How have the costs and outcomes for residents with dementia been assessed in economic evaluations?

## Methods

### Protocol and registration

A protocol for this systematic review was registered with the PROSPERO International Prospective Register of Systematic Reviews on 30 January 2015 (http://www.crd.york.ac.uk/PROSPERO; registration number CRD42015015977).

### Eligibility criteria

Eligible studies included full economic evaluations (e.g. cost-effectiveness analyses, cost-utility analyses, cost-benefit analyses), partial economic evaluations (e.g. cost analyses), and randomised trials reporting more limited information, such as estimates of resource use or costs of interventions, pertaining to structures and processes of care aimed at improving the quality of care for older adults in a residential aged care setting.

*Structures of care* were defined as the workforce and its operations, and included level of staffing, expertise of staff, hours of care per resident per day, and continuity of care. *Processes of care* included activity programs and services implemented in the context of care provision. These definitions were adapted from Donabedian’s quality of care model incorporating structure, process, and outcome [15].

Studies pertaining to interventions that did not apply at a facility or unit level such as individualised pharmaceutical interventions and feeding tubes were excluded from this review.

### Search and study selection

Eight electronic bibliographic databases were searched from inception to the 8th October 2014, including AgeLine, CINAHL, Econlit, Informit (databases in Health; Business and Law; Social Sciences), Medline, ProQuest, Scopus, and Web of Science. An update search was run on 14 December 2015.

The search strategies were developed and reviewed with the assistance of two Health Sciences Librarians with expertise in systematic reviews. The strategy combined terms relating to nursing homes, economics, and older people, limited to English language. No study design or date limits were imposed on the search. The full search strategy is available on PROSPERO.

Due to the large number of results retrieved when searching the multidisciplinary database ProQuest, results were limited to scholarly journals, reports, dissertations and theses, conference papers and proceedings, and working papers. Newspapers, trade journals, wire feeds, magazines, other sources, books, and encyclopedias and reference works were excluded.

Titles and abstracts of studies retrieved were reviewed in full by the primary review author. A second reviewer independently screened 10% of the titles and abstracts. The overall agreement was then calculated using Cohen’s kappa statistic [16]. Full text reports were retrieved for all citations that appeared to meet the inclusion criteria, or where there was any uncertainty. All full text reports retrieved were reviewed independently by two review authors. Disagreement or uncertainty was resolved through discussion and consultation with a third review author. Reasons for excluding studies were documented.

### Data extraction

The Joanna Briggs Institute Data Extraction Form for Economic Evaluations was used to extract data from the included studies [17]. The primary review author extracted all data. Neither the study selection nor the data extraction was blinded.

Data items extracted included descriptive data about the study and analysis including (i) study population/participants, intervention, comparator(s) and outcomes; (ii) study methods including prices and currency used for costing, time period, sensitivity analyses and measures of resource use; (iii) study context (geographical, health care and broader service delivery setting and culture); (iv) analysis methods.

Results for the resource use and/or cost and/or cost-effectiveness measures and the author conclusions were also extracted.

### Risk of bias assessment

Critical appraisal of studies was undertaken using the Joanna Briggs Institute Critical Appraisal Checklist for Economic Evaluations [17], adapted from the Drummond checklist [18], which addressed: the study question; description of alternatives; identification of costs and outcomes; establishment of clinical effectiveness; accuracy, credibility and timing of costs and outcomes; incremental analysis; sensitivity analyses; and generalizability. The appraisal was conducted by the primary review author and ratified by a second reviewer.

### Data synthesis

Data extracted from included studies were analysed and synthesized in a narrative summary to address the stated review objectives. No meta-analysis was conducted due to significant heterogeneity of service configurations in the included studies.

## Results

### Study selection

**Fig. 1:**
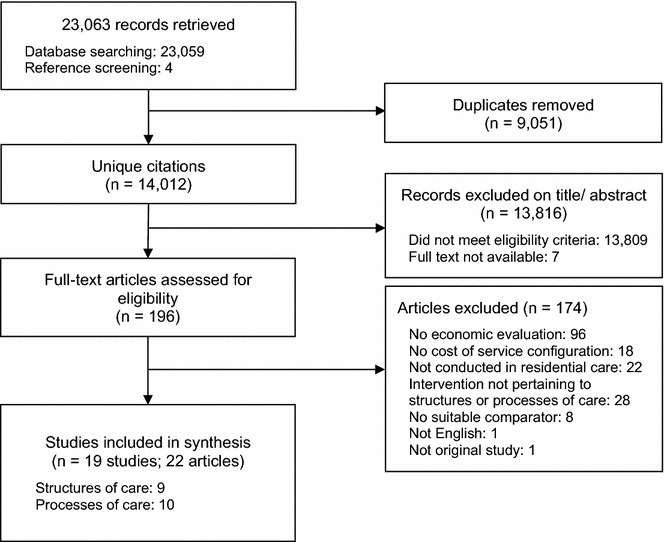
Flow diagram of study selection

The study selection process is presented in Fig. 1. The electronic database search yielded a total of 23,059 citations; an additional 4 citations were identified through searches of reference lists of included studies. A total of 14,012 unique citations were identified after duplicate removal. Full text reviews were conducted for 196 articles and 19 studies, from 22 publications, met the inclusion criteria. The chance-corrected agreement between the abstracts selected by the primary and secondary reviewers was almost perfect with a kappa statistic of 0.88 [19].

### Overview of studies

Table 1 presents the characteristics of studies included in the review. Of the 19 studies included in the review, 12 contained a partial economic evaluation in the form of a cost analysis. Seven studies conducted full economic evaluations, including three cost-benefit analyses, two cost-effectiveness analyses, one cost-utility analysis, and one cost-minimisation analysis. Approximately half of included studies (10/19) were evaluated from an institutional perspective, and only costs occurring within the facility itself were considered. Three studies were evaluated from a health care perspective, with resource use and costs calculated for items such as drugs, hospitalisations and outpatient visits. Four studies were evaluated from a societal perspective, which implies that wider costs for resources consumed in all relevant sectors such as the residential facility, the heath care sector, and by the residents and family members themselves were taken into account. One study took a health and social services perspective, which included resources consumed in the health care sector as well as social services such as audiology, chiropody, and speech therapy. Two studies took the perspective of the insurance providers, including health insurance and long-term care insurance.

**Table 1:** Characteristics of included studies

Source, country	Intervention/comparator	Facility n	Participant n	Study design	Type of economic evaluation; analytic viewpoint	Time horizon	Date/source/currency of economic data	Dementia specific	Setting	Economic outcome
*Structures of care*										
Dorr et al. [20], USA	Registered Nurse (RN) direct care time per resident per day: 30–40 min Less than 10 min	82	1376	Retrospective cohort study	Cost-benefit analysis; societal; institutional	1 year	2001; Secondary sources including national databases, with true costs obtained where possible; *USD*	No	NH	Annual net societal benefit of $3191 per resident per year in nursing home units with 30–40 min of RN direct care time per resident per day compared to less than 10 min
Grabowski and O’Malley [ 34], USA	Off-hours physician coverage via telemedicine vs. on-call physician	11	N/A	Cluster randomised controlled trial	Cost-benefit analysis; insurance provider (medicare)	2 years	Oct 2009–Sep 2011; Estimated cost of hospitalizations to Medicare from recent literature; *USD*	No	NH	15.1 hospitalisations avoided. Net savings of $120,000 per facility per year
Jenkens et al. [36], USA	Green House model Usual care	7	N/A	Cross-sectional	Cost analysis; institutional	N/A	2009; Wages derived from salary.com and payscale.com with 5% increase applied to Green House CNA wage; *USD*	No	SNF	GH facilities use 1.97–2.49% more staff than traditional nursing homes
Maas et al. [23]; Swanson et al. [38]; Swanson et al. [44], USA	Special care unit Traditional unit	1	44	Prospective cohort study	Cost analysis; health care	1 year	Date not disclosed; Resource use measured and unit costs assigned—source of unit cost data not disclosed; *USD*	Yes	NH	Costs of care for residents with dementia in special care units were 29% higher than cost of care on traditional units
Mehr and Fries [24], USA	Special care unit Traditional unit	177	6663	Cross-sectional	Cost analysis; institutional	N/A	Date not disclosed; Resource use data from the resident status measure database, a preliminary version of the national nursing home minimum Data set; *USD*	Yes	NH	Unadjusted resource use was 18% lower on SCUs than other units in the facility; when adjusted for case mix no significant difference in resource use was found
Przybylski et al. [28], CAN	Physical Therapy (PT) & Occupational Therapy (OT) staffing levels: 1.0 FTE PT and 1.0 FTE OT per 50 beds 1.0 FTE PT and 1.0 FTE OT per 200 beds	1	115	Randomised controlled trial	Cost analysis; institutional	2 years	1993/1994; Direct care nursing costs calculated based on the Alberta resident classification system (case mix measure) which estimates average amount of nursing care required per category. Source of wage data not disclosed; *Currency not disclosed.*	No	NH	PT/OT delivered at a 1:50 ratio was more effective at promoting, maintaining, or limiting decline in functional status. The resulting reduction in required care delivery resources was estimated to provide an annual cost saving of $283 per bed (a 1% cost reduction)
Schneider et al. [35], GBR	1.0 FTE occupational therapist Usual care	8	190	Non-randomised experimental trial	Cost analysis; health and social services	1 year	2002–2003; Published unit costs, inflated to 2005; *GBP*	No	CH	Intervention group showed a significant increase in the likelihood of using social services. At 2005 levels, net cost of providing occupational therapy was £16 per resident per week
Sharkey et al. [37], USA	Green House model Usual care	27	240	Cross-sectional	Cost analysis; institutional	N/A	2008–2009; Observational, interview, and survey methods at participating facilities; *N/A*	No	SNF	Total staffing time (excluding administration) in Green House facilities was 18 min less per resident per day that traditional facilities. CNAs in Green House facilities spent 24 min per resident per day more time in direct care activities than CNAs in traditional facilities
Teresi et al. [29], USA	Implementation of an evidence-based education and best practice program: Training staff vs. training staff and nursing home inspectors vs. usual training	45	N/A	Quasi-experimental	Cost-benefit analysis; Societal	2.5 years	2008; Aggregate cost data based on local estimates and published literature; *USD*	No	NH	Training staff was associated with a 15% reduction in annual falls, while training staff and inspectors was associated with a 10% reduction in falls. Range of estimates for the cost-benefit analysis is between a net loss of $26,000 and a net savings of $52,000
*Processes of care*										
Chenoweth et al. [39], AUS	Person-centred care (PCC) Person-centred environment (PCE) Both PCC + PCE Usual care	38	601	Cluster randomised controlled trial	Cost analysis; institutional	8 months	2009–2011; Resource use measured and unit costs assigned using market rates; *AUD*	Yes	RACF	PCC: 7169 per home; PCE: 9198 per home; PCC + PCE: 22,857 per home. Reduced agitation and improvements in resident quality of life for care homes which instituted PCC and PCE. The PCC + PCE intervention produced significant improvements in quality of care interactions and care responses, but no improvements in agitation or quality of life
Chenoweth et al. [31]; Norman et al. [32], AUS	Person-centred care (PCC) Dementia-care mapping (DCM) Usual care	15	289	Cluster randomised controlled trial	Cost-effectiveness analysis; institutional	8 months	2008; Pharmaceutical costs: Australian pharmaceutical benefit schedule Training costs: Bradford University, UK Staff costs: Commonwealth Government Aged Care Nurses’ Award; *AUD*	Yes	RACF	Dementia care mapping was found to be a more expensive and less effective intervention than person-centred care. The cost per negative behaviour averted in the person-centred care group was $8.01 post-intervention and $6.43 at follow-up relative to usual care
MacNeil Vroomen et al. [22], NED	Multidisciplinary Integrated Care (MIC) Usual care	10	301	Cluster randomised controlled trial	Cost-effectiveness analysis; societal	6 months	2007; Health care utilisation collected via patient/proxy interview and medical records. Source of cost data not disclosed. CPI figures sourced from the Dutch bureau of statistics; *EUR*	No	RH	For functional health and QALYs, multidisciplinary integrated care was not found to be cost-effective compared to usual care. For patient-related quality of care, the probability that the intervention was cost-effective compared to usual care was 0.95 or more for ceiling ratios greater than €129
Molloy et al. [25], CAN	Advance Directive program Usual care	6	1292	Cluster randomised controlled trial	Cost analysis; health care	1.5 years	Date not disclosed; Unit prices sourced from local and provincial fee schedules; *CAD*	No	NH	Intervention nursing homes reported 44% fewer hospitalisations per resident (0.27 versus 0.48), and 33% less resource use ($3490 versus $5239) than the control facilities.
Müller et al. [33], DEU	Multifactorial fracture prevention program Usual care	N/A	N/A	Markov-based simulation model	Cost-utility analysis; insurance provider	20 years	2012; Retrospective dataset of costs for NH residents from an insurance fund (n = 60,091), a public German dataset for fracture treatment costs, and catalogue of non-physician care for physical therapy costs; *EUR*	No	NH	Base-case analysis of multifactorial fall prevention resulted in a cost-effectiveness ratio of €21,353 per QALY
Ouslander et al. [43], USA	INTERACT II tools (Interventions to Reduce Acute Care Transfers)	36	N/A	Controlled before-and-after	Cost analysis; institutional	6 months	2010; Wages based on national data; *USD*	No	NH	Intervention group reported 17% reduction in hospitalisation rates. The average cost of the 6-month intervention was $7700 per facility
Paulus et al. [21], NED	Integrated care Traditional care	2	342	Quasi-experimental	Cost analysis; societal	1.2 years	Date not disclosed; Activity based costing, data obtained from participating nursing homes and a published guide for cost research; *EUR*	No	NH	Integrated care had 31% lower informal direct care costs per resident. Total average costs per resident were on average 4% higher in integrated care than traditional care
Rantz et al. [26], USA	Multilevel intervention with expert nurses vs. monthly info packs on ageing and physical assessment	58	N/A	Cluster randomised controlled trial	Cost analysis; institutional	2 years	Date not disclosed; Medicaid cost reports; *USD*	No	SNF	Total costs per resident per day increased 6% in the intervention group, and decreased 3% in the control. The intervention demonstrated improvements in quality of care, pressure ulcers and weight loss
Rovner et al. [27], USA	A.G.E. dementia care program (activities, medication guidelines, educational rounds) vs. usual care	1	81	Randomised controlled trial	Cost analysis; institutional	6 months	Date not disclosed; Monthly billing records; *USD*	Yes	ICF	At 6 months, intervention residents were more than 10 times more likely to participate in activities than controls. Additional cost of the intervention was $8.94 per resident per day
van de Ven et al. [30], NED	Dementia-care mapping (DCM) Usual care	11	318	Cluster randomised controlled trial	Cost-minimisation analysis; health care	1.5 years	2010–2012; Data collected over a period of 18 months. Sources included the Dutch manual of health care cost, and cost prices delivered by a pharmacy and a nursing home; *USD (EUR 1.00* = *USD 1.318)*	Yes	NH	No significant effect on total costs for the intervention

*Countries AUS* Australia; *CAN* Canada; *CHE* Switzerland; *DEU* Germany; *GBR* United Kingdom; *NED* Netherlands; *USA* United States

*Settings CH* care home; *ICF* intermediate care facility; *SNF* skilled nursing facility; *NH* nursing home; *RACF* residential aged care facility; *RH* residential home

Ten (53%) of the included studies were conducted in the United States, three in the Netherlands, two in Canada, two in Australia, one in Germany, and one in the United Kingdom. Ten of the studies involved interventions pertaining to processes of care, while nine examined structures of care. Six studies identified examined dementia-specific service configurations.

Study designs were varied. The most frequent study design was a cluster-randomised controlled trial (7/19), followed by cross-sectional (3/19), randomised controlled trial (2/19), and quasi-experimental (2/19). Other study designs included controlled before-and-after, non-randomised experimental trial, prospective cohort, retrospective cohort, and a Markov simulation model.

The number of participating facilities per study ranged from 1 to 177 (mean: 30; median: 11). Thirteen of the studies recruited resident participants, with sample sizes ranging from 44 to 6663 (mean: 912; median: 301), while five studies assessed facility-level data only.

### Risk of bias

Table 2 presents the results of the assessment of methodological quality of the included studies. The methodological quality of included studies was varied. Some notable deficiencies were found in two of the four studies which indicated their analysis was undertaken from a societal viewpoint. A societal viewpoint is the broadest perspective that can be taken for an economic evaluation and resources consumed in all relevant sectors should ideally be captured using this approach. In an evaluation of enhanced Registered Nurse time, costs beyond the aged care facility e.g. informal carer time or social services consumption were excluded [20]. In a study evaluating the integration of residents with care staff via increased participation in daily activities (e.g. cooking), Paulus and colleagues [21] included costs for formal (staff) and informal (family and friends) care time, but did not include other relevant costs such as medications or hospitalisations.

**Table 2:** Critical appraisal results for included studies using the JBI critical appraisal checklist for economic evaluations

Source	Q1	Q2	Q3	Q4	Q5	Q6	Q7	Q8	Q9	Q10	Q11
	Well-defined question	Comprehensive description of alternatives	All important and relevant costs and outcomes identified	Clinical effectiveness established	Costs and outcomes measured accurately	Costs and outcomes valued credibly	Costs and outcomes adjusted for differential timing	Incremental analysis of costs and consequences	Sensitivity analyses conducted	Study results include all issues of concern to users	Results are generalizable
Chenoweth et al. [31]; Norman et al. [32]	Yes	Yes	Yes	Yes	Yes	Yes	N/A	Yes	Yes	Yes	Yes
Chenoweth et al. [39]	Yes	Yes	No	No	Unclear	Yes	N/A	No	No	No	Unclear
Dorr et al. [20]	Yes	Yes	No	Yes	No	Yes	N/A	No	Yes	No	Yes
Grabowski and O’Malley [34]	Yes	Yes	Yes	Yes	No	Unclear	No	No	No	No	Unclear
Jenkens et al. [36]	Yes	Yes	Yes	No	Yes	Yes	N/A	No	No	Yes	Yes
Maas et al. [23]; Swanson et al. [38]; Swanson et al. [44]	Yes	Yes	Yes	No	Unclear	Unclear	N/A	No	No	No	Unclear
MacNeil Vroomen et al. [22]	Yes	Yes	Yes	Yes	Unclear	Yes	N/A	Yes	Yes	Yes	Unclear
Mehr and Fries [24]	Yes	Yes	Yes	No	Yes	Unclear	N/A	No	No	Yes	Unclear
Molloy et al. [25]	No	Yes	Yes	No	Yes	Unclear	No	No	No	Yes	Unclear
Müller et al. [33]	Yes	Yes	Yes	Yes	Yes	Yes	Yes	Yes	Yes	Yes	Yes
Ouslander et al. [43]	No	Yes	Yes	Yes	Yes	Yes	N/A	No	No	Yes	Unclear
Paulus et al. [21]	Yes	Yes	No	No	Yes	Unclear	Unclear	No	No	No	Unclear
Przybylski et al. [28]	Yes	Yes	Yes	Yes	Unclear	Unclear	No	No	No	Yes	Unclear
Rantz et al. [26]	Yes	Yes	No	Yes	No	Unclear	No	No	No	No	No
Rovner et al. [27]	Yes	Yes	Yes	Yes	Unclear	Unclear	N/A	No	No	Yes	Unclear
Schneider et al. [35]	No	Yes	Yes	No	Yes	Yes	N/A	No	No	Yes	No
Sharkey et al. [37]	Yes	Yes	Yes	No	Yes	Yes	N/A	No	No	No	Unclear
Teresi et al. [29]	Yes	Yes	No	Yes	No	Unclear	Unclear	No	Yes	No	No
van de Ven et al. [30]	No	Yes	Yes	Yes	Yes	Unclear	No	No	No	Unclear	Unclear

In a study evaluating a multidisciplinary integrated care model, MacNeil Vroomen and colleagues [22] also chose a societal viewpoint. This study provides an example of a well-conducted robust analysis that captures all relevant resource use items and costs incurred in all relevant sectors including general practitioner, physical therapy, psychosocial therapy, medical specialists, admission to hospital, informal care, as well as intervention-specific implementation costs.

In terms of the reporting of resource use and costs there were notable deficiencies in a number of studies. Six out of 19 of the included studies did not disclose the date for their cost data collection [21, 23–27]. Three studies did not disclose the source of their cost data [22, 23, 28], and one study also failed to disclose the currency used in the analysis [28]. There were also deficiencies in the source of cost data in two studies [29, 30]. In a study of dementia-care mapping, Van de Ven and colleagues [30] calculated nursing home staff costs for their analysis of 11 nursing homes based on the gross costs of a single nursing home. In this scenario, it is unclear whether the costs from a single facility can reliably be generalised to the 11 nursing homes which were included in the study. In an implementation study of evidence based education, Teresi and colleagues [29] were unable to obtain site-specific data for the 45 facilities that participated. Aggregated local estimates combined with cost data from published literature were utilised in lieu of site-specific data, which may not have been representative of the facilities included in the analysis.

Five studies conducted sensitivity analyses [20, 22, 29, 31–33]. Eight studies were undertaken over a time horizon greater than one year [21, 25, 26, 28–30, 33, 34], of which one study made adjustments for differential timing of costs over the study period [33].

### Structures of care

Table 3 provides a summary of the economic results reported in studies pertaining to structures of care.

**Table 3:** Summary of results pertaining to structures of care

Intervention	Source	Effectiveness	Cost	Randomised design	Key findings
Enhanced staffing levels					
30–40 min of RN direct care time per resident per day vs. less than 10 min	[20]	+	–	No	Enhanced staffing levels have the potential to create cost savings from a societal perspective Increasing nurse staffing in nursing homes demonstrated net reduction in re-hospitalisation, pressure ulcer presence, and urinary tract infections Enhanced PT and OT services delivered improved functional status and reduced nursing costs Occupational therapy has the potential to reduce secondary care costs including hospitalisation, and may uncover unmet needs for services
Physical therapy and occupational therapy (PT/OT) staffing levels: 1:50 vs. 1:200	[28]	+	–	Yes	
1.0 FTE occupational therapist vs. usual care	[35]	+	+	No	
Off-hours physician coverage via telemedicine vs. on-call physician	[34]	+	–	Yes	Facilities accessing off-hours physician coverage via telemedicine had fewer resident hospitalisations than those facilities who did not utilise the telemedicine program or those who only had access to an on-call physician
Staffing configurations in specialised models of care					
FTE comparisons in Green House model vs. traditional institutional care	[36]	None	+	No	Green house facilities provide more direct care time to residents compared to traditional units/facilities There is an increase in direct care FTEs, which is offset by a reduction in administration and support staff FTEs
Direct care time in Green House vs. traditional skilled nursing facilities	[37]	+	–	No	
Special care unit (SCU) vs. traditional unit	[23, 38, 44]	±	+	Yes	Costs of care are higher on SCUs and in SCU facilities, than non-SCU facilities Special care units provide more direct care time to residents compared to traditional units/facilities
SCUs vs. traditional units in SCU facilities SCU facilities vs. non-SCU facilities	[24]	None None	0 +	No	
Staff education					
Implementation of an evidence-based education and best practice program vs. usual training	[29]	+	+	Yes	Evidence-based education programs show potential to reduce falls compared to non-evidence-based training The potential for cost savings is highly dependent on the true cost of falls

*Effectiveness +* intervention provides greater health benefit than comparator; *0* intervention provides equivalent health benefit to comparator; *−* intervention provides lower health benefit than comparator

*Cost +* intervention costs are higher than comparator; *0* intervention costs are equal to comparator; *−* intervention costs are lower than comparator

#### Staffing levels

Four studies evaluated the costs and effects of enhanced staffing levels, including increasing the amount of direct nursing care time for each resident [20], employing a full-time occupational therapist [35], increasing the staffing level of both physical and occupational therapists [28], and implementing off-hours physician coverage via telemedicine [34]. Results suggest that enhanced staffing levels, whilst being associated with increases in staffing costs provide the potential for cost savings in other areas. For example, one study found that increasing registered nurse staffing in nursing homes to ensure 30–40 min of direct care time per resident per day reduced the incidence of pressure ulcers, hospitalisations, and urinary tract infection rates resulting in a net societal benefit of US$3191 per resident per year [20]. Similarly, another study reported that increasing the staff to resident ratio for physical therapists and occupational therapists was more effective at promoting, maintaining, or limiting decline in functional status. The resulting reduction in required care delivery resources was estimated to provide an annual cost saving to the institution of $283 per resident [28]. A third study which evaluated the benefit of a full-time occupational therapist reported a significant reduction in secondary health care costs (including hospital admissions) and an increase in the use of social services, though the cost of providing occupational therapy was not offset by the savings in health care [35]. Finally, a fourth study found that increasing the availability of physician care during the off-hours via a dedicated telemedicine service decreased annual hospitalisations by 11.3% annually [34]. Based on an average nursing home size of 113 beds, net savings to US Medicare were estimated to be $120,000 per annum for facilities which utilised the telemedicine service to a greater extent [34].

Another important finding from this review was the assimilation of currently available evidence relating to the costs and effectiveness of staffing levels in specialised models of residential care, including Green House facilities and dementia special care units [23, 24, 36, 37]. Green House facilities provide a small, home-like model of care as an alternative living environment to the traditional skilled nursing facilities in the United States. In the Green House model, ten to twelve residents live in a self-contained residence designed to look and feel like a private home. Dementia special care units (SCUs) are separate units within a residential care facility that have been adapted specifically for people living with dementia.

Three out of four studies which evaluated staffing levels in specialised models of care (Green House facilities and dementia special care units) reported that these types of specialised models generally provided more direct care time to residents compared to traditional facilities [23, 36, 37]. Resource use and cost implications associated with staffing levels in specialised models of care, however, were conflicting across studies with no clear results. With regard to special care units, one study reported no difference in resource use once adjusted for case mix [24], while the other reported higher resource use but made no adjustments for case mix [23]. Of the two studies on Green House facilities, one reported lower staffing requirements than traditional units [37] while the other reported increased staffing requirements of 2.0–2.5% compared to traditional facilities [36]. None of the studies evaluating staffing levels in specialised facilities established clinical effectiveness. Swanson, Maas and Buckwalter [38] did report significant results found with indirect outcome measures in the form of reduced catastrophic reactions and increased social interactions on special care units with the number of reactions decreasing from 156 pre-intervention to 48 at the 12-month follow-up in the SCU group compared to the control group which reported catastrophic reactions of 82 and 46 at pre-intervention and follow-up respectively (p = 0.035).

#### Staff education

One study evaluated the implementation of an evidence based staff education and best practice program targeting ‘vision awareness’ to improve staff knowledge of visual impairments and to reduce the incidence of falls [29]. It was estimated that the intervention resulted in a reduction in the number of annual falls between 5 and 12 in a typical 200-bed nursing home in New York State. Depending on estimates used for the cost of falls, the net societal benefit ranges between a net loss of US$26,000 and a net saving of US$52,000 calculated in 2008 US dollars.

### Processes of care

Table 4 provides a summary of the economic results reported in studies pertaining to processes of care.

**Table 4:** Summary of results pertaining to processes of care

Intervention	Source	Effectiveness	Cost	Randomised design	Key findings
Dementia-specific care					
Person-centred care (PCC) vs. usual care (UC) Person-centred environment (PCE) vs. UC Both (PCC + PCE) vs. UC	[39]	+ + Unclear	+ + +	Yes	Person-centred care has the potential to reduce agitation and aggression in residents living with dementia Disparate implementation methods and mixed findings suggest a need for future research to examine the cost-effectiveness of person-centred care as well as different methods for assessing clinically-relevant quality of life
PCC vs. UC Dementia-care mapping (DCM) vs. UC	[31, 32]	+ +	+ +	Yes	
Dementia-care mapping (DCM) vs. usual care	[30]	0	0	Yes	
A.G.E. dementia care program (activities, medication guidelines, educational rounds) vs. usual care	[27]	+	+	Yes	For an additional cost, activity programs and psychiatric care can reduce behavioural symptoms, antipsychotic medications, and restraints, as well as increase activity participation rates for residents with dementia
Integrated care					
Multidisciplinary Integrated Care model vs. UC	[22]	Unclear	+	Yes	There is limited cost-saving potential for integrated care in nursing homes If there was unmet care, a multidisciplinary integrated model could address this gap; however a trade-off must be made as to whether the additional benefit is worth the additional cost
Integrated care vs. traditional care	[21]	NA	+	No	
Quality improvement initiatives					
Advance Directive program vs. usual care	[25]	0	–	Yes	Activity programs aimed at reducing health care utilisation and hospitalisations have the potential to create cost savings from a broader health care perspective
INTERACT II tools (interventions to reduce acute care transfers)	[43]	+	+	No	
Multifactorial fracture prevention program provided by a multidisciplinary team vs. no prevention in newly admitted nursing home residents	[33]	+	+	No	
Multilevel intervention with expert nurses vs. monthly info packs on ageing and physical assessment	[26]	+	+	Yes	It is possible for facilities in need of quality of care improvements to build the organisational capacity to improve while not increasing staffing or costs of care

*Effectiveness +* intervention provides greater health benefit than comparator; *0* intervention provides equivalent health benefit to comparator; *−* intervention provides lower health benefit than comparator

*Cost +* intervention costs are higher than comparator; *0* intervention costs are equal to comparator; *−* intervention costs are lower than comparator

#### Dementia-specific care

Four studies evaluated dementia-specific care interventions compared to usual care. These interventions included person-centred care implemented through staff training [31, 32, 39] or dementia-care mapping [30, 31], and a dementia care program which aimed to reduce behaviour disorders [27].

Supporting personhood has been identified as a foundation for quality care for people living with dementia [40]. Person-centred care centres on relationships with others and the theory that warm and compassionate care interactions should increase well-being, while disrespectful and disengaged care interactions are thought to lead to decreased well-being and increased agitation [14]. Person-centred care can be implemented at residential care facilities in different ways.

Two methods of implementing person-centred care were evident from the identified studies. One method, which researchers called ‘person-centred care’ involved off-site staff training followed by a period of on-site supervision and telephone support [31, 39]. The second, more resource-intensive method was dementia-care mapping which required selected staff members to become certified through basic and advanced training. The mappers then completed systematic observation of residents with dementia, from which feedback was given to care staff and managers in order to assist with planning, implementation and assessment of person-centred care [30, 31]. Chenoweth and colleagues [31] found that the first method of training and support dominated dementia-care mapping, as their results showed dementia-care mapping to be more expensive and less effective. Van de Ven and colleagues [30] on the other hand, found dementia-care mapping to be a cost-neutral endeavour.

The most common primary outcome assessed in this subgroup was agitation using the Cohen Mansfield Agitation Inventory (CMAI) [30, 31, 39]. Van de Ven [30] and Chenoweth [31] both found that dementia-care mapping had no significant effect on agitation with study follow-up times of 18 and 8 months respectively. Two studies by Chenoweth and colleagues [31, 39] reported small statistically significant decreases in agitation as a result of their person-centred care intervention, with follow up conducted at 14 and 8 months.

Other outcomes assessed (and measurement tools used) across this subgroup included emotional responses in care (ERIC), quality of life (DemQol, DemQol-proxy, Qualidem, EQ-5D, and QUALID), care interaction quality (Quality of Interactions Schedule), psychiatric symptoms (neuropsychiatric inventory), behavioural symptoms (Psychogeriatric Dependency Rating Scale Behaviour Subscale), antipsychotic drug and restraint use, cognition (mini-mental state examination, MMSE), level of nursing care (resource utilisation groups, RUG-II), and activity participation rates. Some small improvements were found in quality of care interactions, resident care responses, and quality of life measured with the DemQol-proxy [39].

Rovner and colleagues [27] evaluated a dementia care initiative consisting of organised ‘day-care’ activities from 10AM-3PM daily, combined with psychotropic medication guidelines, and educational rounds performed by a psychiatrist. In contrast to the person-centred care interventions, the dementia care program was not based exclusively on relationships but was developed to provide structure and stimulation through scheduled activities such as music and games. While the study did not find any cost reductions to offset the intervention costs, the authors did report that intervention residents were over ten times more likely to participate in activities than the comparison group. The intervention was also found to decrease the prevalence of behaviour disorders and the use of antipsychotic drugs and restraints.

#### Integrated care

Two studies evaluating integrated care delivery found higher costs in the intervention group compared to usual care [21, 22]. Integration strategies aim to provide a level of service that is more individualised and sensitive to the personal circumstances of the resident [41], and can be applied to residential care at a number of levels [42].

Paulus and colleagues [21] examined integrated care in the sense of integration between residents and care staff. Residents lived in smaller-scale facilities with increased levels of social activities, more flexibility in daily routines, and the opportunity to engage in daily activities such as cooking, cleaning and laundry. Integrated care was shown to have lower informal care costs (care provided by family and friends) when compared to traditional care, while both the costs of formal care (provided by staff) and total average costs were higher in integrated care.

MacNeil Vroomen and colleagues’ [22] integrated care model focused on the integration of health disciplines through case-conferencing. The intervention included a quarterly assessment of all residents by nursing assistants, multidisciplinary meetings with a primary care physician, nursing home physician, nurse, psychotherapist, and other disciplines involved in resident care, and a multidisciplinary consultation for those residents with more complex health needs. Three outcomes were measured: quality of care, functional health, and quality of life. This study found that for functional health and quality-adjusted life years (utility scores calculated from the SF-6D), integrated care was not cost-effective compared to usual care. However, for patient-related quality of care, the probability that integrated care was cost-effective compared to usual care was 0.95 or more for ceiling ratios greater than €129.

#### Quality improvement initiatives

Four studies conducted facility-level interventions aimed at improving the quality of care [25, 26, 33, 43]. Interventions included an advance directive program to educate and assist residents with a written expression of their wishes to guide family and health care workers in their care choices [25], an intervention to reduce acute care transfers through the early identification, assessment, communication, and documentation of changes in resident status [43], a quality improvement intervention involving monthly visits and support by expert nurses [26], and a fracture prevention program for all residents upon admission to a residential care facility [33]. The advance directive program [25], the intervention to reduce acute care transfers [43], and the multifactorial fracture prevention program [33] were all found to reduce hospitalisation rates, resulting in cost savings from a broader health care perspective. The quality intervention with expert nurses was found to improve quality of care (measured with the Observable Indicators of Nursing Home Care Quality (OIQ) instrument.), and reduce the incidence of pressure ulcers and weight loss [26]. In all four studies, the increased costs associated with implementation of the interventions were borne by the aged care facility.

## Discussion

In comparison with the health care sector, where economic evaluations are common practice for pharmaceuticals and medical technologies, this review identified a paucity of economic evidence relating to the structures and processes of care in the residential aged care sector. A total of 19 studies were identified by this review: 12 cost analyses, one cost-minimisation analysis, one cost-utility analysis, two cost-effectiveness analyses, and three cost-benefit analyses.

Despite the heterogeneity of interventions and outcome measures, synthesis of study results revealed several common themes. Results from three studies suggest a potential for cost savings to the health care sector by increasing the amount of direct care time provided to each resident [20, 28, 35]. Benefits reported were wide ranging from reductions in the frequency of hospitalisations to improved functional status for the residents. The best means of achieving these outcome improvements is unclear, however, as the included studies focused on a disparate array of staff positions including registered nurses, occupational therapists, and physiotherapists. These positive results highlight an opportunity for future research to explore cost-effective methods of increasing the amount of direct care time to residents, and the optimal skill set and configuration of staff (e.g. nurses, allied health professionals, and other aged care workers) needed to achieve the best outcomes for individual residents.

Interestingly, increased levels of direct care time were found in the small, home-like ‘Green House’ model, as well as the dementia special care units. While we would expect to see cross-sectoral benefits (beyond the aged care sector and into the health care sector) similar to those reported in the enhanced staffing interventions, none of the studies actually measured costs in the health care sector. Three of the four did not report any effectiveness measures [24, 36, 37], while the fourth found no effect on cognitive or functional abilities [44]. By not including costs from all relevant sectors, these studies may be underestimating the potential value of specialised care settings.

Another aspect of residential care that was shown to create cost savings from a broader health care perspective were quality improvement initiatives, such as activity programs and interventions aimed at reducing health care utilisation and hospitalisations. While quality improvement initiatives tend to come at a cost to the facility in terms of planning and implementation, the flow-on effects of improving care quality is likely to extend to other areas of health services. Many of these initiatives, however, such as the quality improvement projects evaluated by Ouslander and colleagues [43], and Rantz and colleagues [26], along with more than half of included studies in this review, focused cost analyses on intervention and care costs incurred by the facility only.

The remaining studies are difficult to generalize, largely due to differing implementation methods. In terms of caring for individuals with dementia, recent research into person-centred care suggests its potential to reduce agitation and aggression [31, 39], though this was not a unanimous conclusion [30]. Despite the sound methodological quality of these three studies, disparate implementation methods render it difficult to draw any definitive conclusions. For instance, of the two studies that considered dementia care mapping, one study had two experienced, accredited researchers as well as two care staff from each facility to conduct the mapping [31] while the second study used two care staff from each facility but no researchers [30]. These disparities raise questions about the conclusions drawn, as the two studies described reported higher costs and cost-neutrality respectively.

The concept of integrated care is not well-defined, and is therefore difficult to generalize. Two studies identified by this review defined integrated care in terms of integration between staff and residents [21], and integration across disciplines [22]. Both integrated care interventions reported limited cost-saving potential, however further research in this area is needed which links costs to outcomes. The study of integrated care between staff and residents [21] considered only the costs of care, with no attempt to measure outcomes. The multidisciplinary integrated care method, which conducted full cost-effectiveness analyses, found that for resident-related quality of care, the probability that the intervention was cost-effective compared to usual care was 0.95 or more for ceiling ratios greater than €129, while the same intervention was not cost-effective in terms of functional health or quality adjusted life years.

Another issue affecting the generalizability of findings is the geographic concentration of research in the United States. Research conducted outside of the United States is sparse. More than half of the included studies were conducted in the United States, while the remaining third were split between the Netherlands, Germany, Canada, Australia, and the United Kingdom. While these findings are consistent with a recent systematic review of randomised controlled trials in care homes, which reported that 50% of the randomised controlled trials undertaken in care homes were from the United States [45], they do highlight a need for research in a wider array of countries and health systems to increase transferability of results.

Another important factor to facilitate transferability of findings in residential aged care, and particularly dementia-specific models of care, is the question of the most appropriate primary outcome measure to use in economic evaluation. All of the dementia-specific studies into person-centred interventions used agitation as the primary outcome, and some small but significant decreases were detected for person-centred care and person-centred environments [31, 39]. Agitation is an outcome measure that is specific to dementia interventions, and therefore comparisons across a broader set of service configurations cannot be made. Given finite resources and a limited budget devoted to aged care, additional investment in one program will likely require a reduction or de-investment in another program in order to free up the necessary resources. A broader outcome measure such as a quality of care and/or a quality of life instrument, which is designed to combine a range of outcomes into a single composite outcome, applicable to all aged care residents, would allow decision makers to make comparisons across differing programs. Each of the three studies focused on person-centred interventions incorporated quality of life instruments as secondary outcome measures. Five different instruments were used: QUALID [31], DEMQOL [39], DEMQOL-proxy [39], EQ-5D [30], and Qualidem [30]. However none of the instruments were able to show significant group differences between the intervention and control groups with the exception of the DEMQOL-proxy, which is completed by a family member or carer on behalf of the person with dementia. Further research is needed to identify appropriate and meaningful quality of care and quality of life instruments for residents of residential care homes, particularly those living with dementia or cognitive decline, which allows comparisons to be made at a service planning level.

Acknowledging that the economic evidence of program features which directly relate to how care is provided in terms of the workforce and its operations (structures of care) and the services provided (processes of care) is limited, we have selected a number of recommendations for change based on the best evidence available. Firstly, increasing the amount of direct care time provided to each resident appears to have wide-ranging benefits at both an institutional and health care level. While further research is needed, additional direct care time provided by nurses, allied health professionals, and other aged care workers all appear to provide benefit. Secondly, benefits arising from initiatives such as increased direct care time or quality improvement initiatives are likely to occur in the health care sector rather than the aged care sector. Future research and policy decisions surrounding residential care initiatives should strive to include health care costs and benefits when considering resource allocation decisions.

In terms of methodological recommendations, our primary suggestion is improved transparency in reporting study methods and results. Future economic evaluations in this area should strive to meet the quality standard for reporting economic evaluation as specified in the Consolidated Health Economic Evaluation Reporting Standards (CHEERS) statement [46] including the quantities of resources used in addition to costs and incorporating the measurement and valuation of service outcomes and quality of life. Disclosures should also be included to indicate the timing of cash flows and the sources of cost data. Secondly, we would strongly encourage future economic research in this area to evaluate both costs and effectiveness in the form of a full economic evaluation. The usefulness of studies containing only partial economic evaluations is limited for policy and decision makers, in that they do not present the case on whether the costs of a course of action is worthwhile in terms of benefits provided to improve quality of care. Finally, we recommend that, where possible, future studies incorporate a societal perspective (especially in considering benefits that may occur in the healthcare sector offsetting costs accrued in the provision of social care) in order to better inform decision makers of the true benefit of an intervention.

This systematic review has several limitations. Firstly, the search strategy was restricted to English-language publications, which may have resulted in some relevant international research being excluded. Secondly, due to the large number of results retrieved when searching the multidisciplinary database ProQuest, limits to source type were applied that were not part of the original search strategy. The ProQuest search was limited to scholarly journals, reports, dissertations and theses, conference papers and proceedings, and working papers. Newspapers, trade journals, wire feeds, magazines, other sources, books, and encyclopedias and reference works were excluded. While this may have resulted in some relevant research being missed, this limitation was justified to maintain the feasibility of abstract screening within the given time constraints. Finally, due to the broad scope of this review, the synthesis and analysis of results was limited by the heterogeneity of included studies.

## Conclusions

This review provides the first comprehensive summary of the existing economic evidence pertaining to workforce structures and care processes in residential care, and highlights an urgent need for robust economic evaluations to inform future service development in this area. In order to fully capture the impact of an intervention or model of care in a residential aged care setting, it is important to take a societal perspective when conducting economic evaluations. The inclusion of broader health care costs in economic evaluations of interventions in residential care, in particular the use of hospitals, is critical for ensuring the value of the intervention is not underestimated. Furthermore, the practical application and transferability of findings would benefit from identifying appropriate and meaningful outcome measures that can be used at a service planning level.

This review also brings to light the potential value of direct care time for residents in care homes. Future research should explore cost-effective methods for increasing the amount of direct care time to residents, and identification of the most appropriate skill mix (with comparison between nurses, allied health professionals, and other aged care workers) for the provision of care according to the care needs of the individual.

Economic evidence is essential to the promotion of efficiency, facilitating future policy directions within the aged care sector and will assist in identifying and quantifying the cross-sectoral impacts of new innovations in the structures and processes of care in terms of both the costs and benefits provided.

## Abbreviations

CMAI: Cohen Mansfield Agitation Inventory
DEMQOL: dementia health-related quality of life
EQ-5D: EuroQol-5 dimensions
PRISMA: preferred reporting items for systematic reviews and meta-analyses
QUALID: quality of life in late-stage dementia
SCU: special care units
SF-6D: short form 6 dimensions
